# Unusual T-Lymphoblastic Blast Phase of Chronic Myelogenous Leukemia

**DOI:** 10.1155/2014/304359

**Published:** 2014-06-24

**Authors:** Jie Xu, Shaoying Li

**Affiliations:** ^1^Department of Pathology, University of Alabama at Birmingham, Birmingham, AL 35294, USA; ^2^Department of Pathology, Microbiology, and Immunology, School of Medicine, Vanderbilt University, 4605 TVC, Nashville, TN 37232-5310, USA

## Abstract

T-lymphoblastic leukemia/lymphoma (T-ALL) presenting as blast phase of chronic myelogenous leukemia (CML-BP) is rare. In patients without history of CML, it is difficult to differentiate between CML-BP or de novo T-ALL. Here we reported 2 unusual cases of T-ALL presenting as CML-BP. Case 1 was a 24-year-old female with leukocytosis. Besides T-lymphoblasts (32%), her marrow exhibited some morphologic features of CML. Multiple remission or relapsing marrow had never demonstrated morphologic features of CML. Despite of imatinib treatment and stem cell transplant, she died 2.5 years later. Case 2, a 66-year-old male with diffuse lymphadenopathy, showed T-ALL in a lymph node and concurrent CML chronic phase (CML-CP) in his marrow. Same BCR-ABL1 fusion transcript with minor breakpoint was present in both the lymph node and marrow specimens. Although both cases did not have a history of CML, both cases represented T-lymphoblastic CML-BP with unusual features: Case 1 is unusual in that it presented as T-ALL with some CML morphologic feature but never showed CML-CP in her subsequent marrows biopsies; Case 2 is the first reported case of T-lymphoblastic CML-BP harboring BCR-ABL1 transcript with a minor breakpoint.

## 1. Introduction

Chronic myelogenous leukemia (CML) is a myeloproliferative neoplasm originating from abnormal pluripotent bone marrow stem cells. In greater than 95% of CML, there is a consistent presence of* BCR-ABL1* fusion gene located in the Philadelphia (Ph) chromosome resulting from chromosomal translocation t(9; 22)(q34;q11.2). The disease course of CML is typically divided into 3 phases: chronic phase (CP), accelerated phase, and blast phase (BP) [1]. CML-BP resembles an acute leukemia and is characterized by the presence of at least 20% blasts in peripheral blood or bone marrow or extramedullary infiltration by blasts. In addition to Ph chromosome, blast phase transformation of CML is usually accompanied by additional chromosomal abnormalities, such as +8, +Ph, i(17q), +19, −Y, +21, +17, or monosomy 7, suggesting that CML-BP is the evolution of a* BCR/ABL1*-positive clone [1, 2]. The blasts in CML-BP usually have myeloid phenotype, representing 70% of the cases. Approximately 30% of cases demonstrate lymphoblastic phenotype which is predominantly B-cell lineage [3, 4]. T-lymphoblastic BP of CML is very rare and so far approximately 50 cases have been reported in the English literature [5–9].

*BCR-ABL1* fusion gene, the hallmark of CML resulted from t(9;22), is also present in 25% of adults ALL and 2–4% of childhood ALL. Three different breakpoint clusters have been described in BCR, which correspond to different sized BCR-ABL1 fusion proteins. The m-BCR (minor) in intron 1 or 2 encodes the e1a2 fusion transcript and the corresponding p190 protein, which is prevalent in ALL. The M-BCR (major) encodes the e13a2 and/or e14a2 transcripts encoding the p210 protein present in 95% of CML. The *μ*-BCR (micro), which encodes e19a2 transcript and the corresponding p230 protein, is rare and associated with CML with prominent neutrophilic maturation.

T-ALL comprises about 25% of adult ALL and 15% of childhood ALL. It is more common in adolescents than in younger children with a slight male predominance. Patients often present with a high leukocyte count and a large mediastinal mass or other tissue masses [10].* BCR-ABL1* fusion gene is relatively common in B-ALL (20–30% of all cases) [11], but very rare in T-ALL [12]. In a childhood ALL survey, T-ALL was found in 9% of all Ph positive cases, indicating an overall frequency of less than 0.3% in ALL patients [13]. The presence of* BCR-ABL1* in ALL indicates a worse prognosis.

Since T-lymphoblastic BP of CML can mimic* BCR-ABL1*-positive T-ALL, it is very challenging to differentiate these two entities in a patient who does not have a previous or concurrent history of CML. Here we reported 2 unusual cases who had no history of CML and presented as T-lymphoblastic leukemia with t(9;22), raising the differential diagnosis of T-lymphoblastic BP of CML versus* BCR-ABL1*-positive T-ALL.

## 2. Case Reports

### 2.1. Case 1

**Figure 1:**
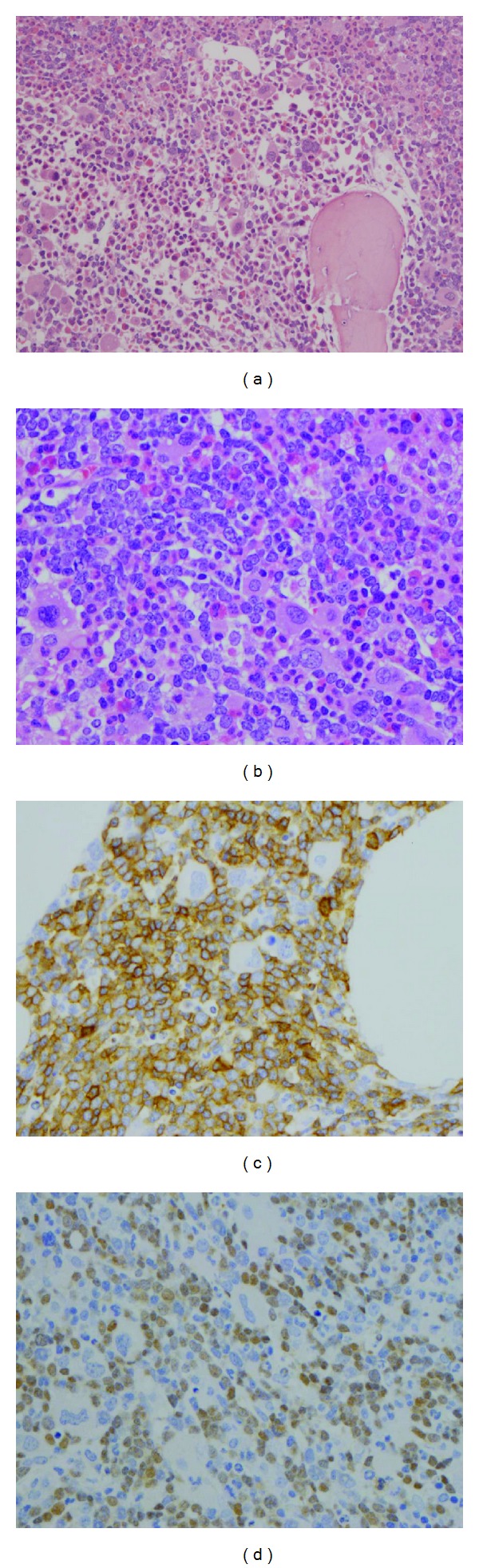
Bone marrow biopsy of Case 1. The bone marrow demonstrated morphologic features of chronic myelogenous leukemia and increased blasts ((a) H&E: 200x; (b) H&E: 400x) with a T-lymphoblastic immunophenotype ((c) CD5: 400x; (d) TdT: 400x).

The patient was a 24-year-old female with leukocytosis. The white blood cell count was 187,900/*μ*L with 10.5% blasts, the hemoglobin was 9.7 g/dL, and the platelet count was 281,000/*μ*L. In addition to the significant number of circulating blasts, her peripheral blood smear showed markedly increased granulocytes with a left-shifted maturation and mild basophilia (WBC differential: Blasts: 10.5%, small to intermediate in size; myelocytes: 4%; metamyelocytes: 2.5%; neutrophils: 57%; eosinophils: 4%; basophils: 3%; monocytes 4%; lymphocytes: 15%). Bone marrow biopsy (Figure 1) showed marked myeloid hyperplasia with left shift, hyperplasia of small monolobated megakaryocytes, and increased blasts (32%). Flow cytometry of the peripheral blood and bone marrow showed identical T-lymphoblastic phenotype: positive for CD45, bright cytoplasmic (cy) CD3, CD1a, CD2, CD5, CD7, CD4, CD8, CD34, and CD38. This lymphoblast population did not express CD19, cy-CD22, CD33, CD15, and cy-MPO. Immunohistochemical stains further confirmed the T-lymphoblastic lineage: CD3+, CD5+, and TdT+ (Figure 1). Myeloid blasts were less than 1% in both peripheral blood and bone marrow. Cytogenetic analysis demonstrated an abnormal female karyotype: 46, XX, t(9;22)(q34;q11.2)[18]/46, idem, del(7)(p15)[2]. FISH study revealed* BCR-ABL1* rearrangement and RT-PCR showed the* BCR-ABL1* fusion gene involving the major breakpoint (encoding the p210 protein) with a* (BCR-ABL1)/BCR* ratio of 0.15.

After receiving Hydrea, Hyper-CVAD, and imatinib, she reached clinical and morphologic remission but low level persistent disease by quantitative RT-PCR with* (BCR-ABL1)/BCR* ratio of 0.02–0.05. She relapsed 14 months later and was treated with Vincristine, Cytarabine, Mitoxantrone, dexamethasone, and dasatinib. Again she reached morphologic but not molecular remission. Then she underwent an ablative match-unrelated donor transplant. Three months after transplant, she had the 2nd relapse and died (2.5 years after the initial diagnosis). Different from her diagnostic marrow biopsy, her relapsed marrow biopsies only demonstrated T-ALL but never showed any morphologic features of CML and her remission marrow biopsies never demonstrated any morphologic features of CML-CP.

### 2.2. Case 2

**Figure 2:**
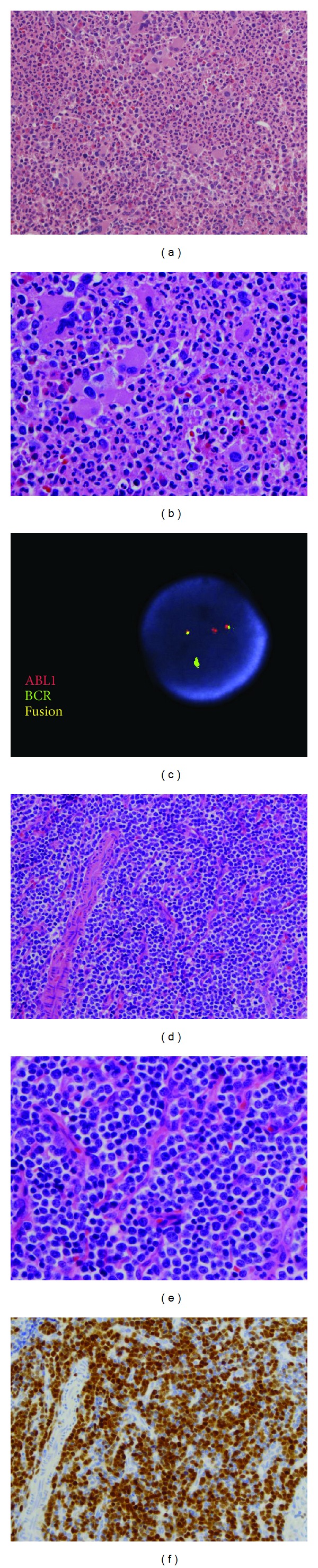
Bone marrow and lymph node biopsies of Case 2. The bone marrow demonstrated typical morphology of chronic myelogenous leukemia, chronic phase ((a) H&E: 200x; (b) H&E: 400x) and FISH showed* BCR/ABL1* fusion (c); lymph node demonstrated lymphoblastic lymphoma morphology ((d) H&E: 200x; (e) H&E: 400x) with a T-lymphoblastic immunophenotype by flow cytometry (not shown) and was positive for TdT ((f) immunohistochemistry: 200x).

The patient was a 66-year-old male with diffuse lymphadenopathy and B symptoms. The white cell count was 56,800/*μ*L (WBC differential: Neutrophils: 70.0%; Eosinophils: 2.0%; Basophils: 2.0%; Metamyelocytes: 1.0%; Myelocytes: 7.0%; Monocytes: 11.0%; Lymphocytes: 3.0%), the hemoglobin was 9.9 g/dL, and the platelet count was 314,000/*μ*L. As the differential demonstrated, his peripheral blood showed increased left-shifted granulocytes, mild basophilia, and absolute monocytosis. There are no circulating blasts. Bone marrow biopsy showed a hypercellular marrow (95–100% cellularity) with marked myeloid hyperplasia and left-shift, increased hypolobated megakaryocytes, and no increase in blasts (Figure 2). Flow cytometry of bone marrow showed no increased myeloid cells (1% blasts) and no T lymphoblasts were present. The concurrent left inguinal lymph node biopsy revealed an effaced lymph node with diffuse infiltration of small to intermediate sized blasts (Figure 2). These blasts were CD3+, CD1a+, and TdT+ (Figure 2) by immunohistochemistry. Flow cytometry of the lymph node revealed a population of T-lymphoblasts which were CD45+, CD1a+, CD2+, sCD3−, cy-CD3+, CD4−, CD5+, CD7+, CD8−, CD10−, CD19−, CD20−, CD38+, CD16−, CD57−, CD103−, CD13−, CD33+dim, CD34−, CD117+/−, and TdT+. Cytogenetic analysis showed an abnormal karyotype in the bone marrow: 46,XY,t(9;22)(q34.1;q11.2)[12]/47,idemdel(5)(q13q33),+MAR[CP8], and a complex karyotype in the lymph node: 46,XY,del(5)(q13q33),t(9;22)(q34;q11.2),add(12)(p11.2),+14,−22[11]/51,idem,+8,+10,+11,+13,+19[9]. FISH studies were positive for* BCR-ABL1* in both peripheral blood and bone marrow (Figure 2), but negative for rearrangements of the FIPIL1/PDGFRA. RT-PCR showed a* BCR-ABL1* fusion transcript with minor breakpoint encoding the p190 protein.

The patient was diagnosed as CML-CP with monocytosis in the bone marrow and T-ALL BP of CML in the lymph node. His monocytosis (11%) in the peripheral blood was consistent with the minor BCR breakpoint [14]. After being treated with cyclophosphamide, daunorubicin, vincristine, and prednisone and imatinib, he was lost to follow up.

## 3. Discussion

Most CML cases (~85%) are diagnosed during the chronic phase, whereas some CML patients present with CML-BP. The nature of the blasts in CML-BP is critical in directing therapy decisions.

T-lymphoblastic BP of CML is very rare and only limited number of cases have been reported. The patients are usually adults with a male predominance. Most cases have a history of CML or concurrent CML in bone marrow. T-lymphoblastic BP of CML predominantly involves extramedullary sites including lymph nodes (most frequently), liver, spleen, and mediastinum. The t(9:22) can be the sole cytogenetic abnormality or as a part of a complex karyotype. In all of the previously reported cases of T-lymphoblastic CML-BP,* BCR-ABL1* gene fusion occurs at the* BCR* major breakpoint with a protein product of p210. Most patients showed a poor prognosis.

In patients presented with a* BCR-ABL1* positive T-ALL but without previous history of CML, the differential diagnosis between de novo T-ALL and T-lymphoblastic BP of CML can be challenging. In contrast to* BCR-ABL1*-positive B-ALL,* BCR-ABL1*-positive T-ALL is extremely rare with <30 cases reported. Raanani et al. reviewed the literature and summarized some helpful features for the differential diagnosis [8]. Features that support a diagnosis of* BCR-ABL*-positive T-ALL include young age (children), lack of previous history or concurrent CML, BM involvement, minor* BCR* breakpoint, and TCR rearrangement. On the other hand, previous history or concurrent CML, extramedullary involvement with T lymphoblasts but no marrow involvement, and absence of TCR gene rearrangement will support the diagnosis of T-lymphoblastic BP of CML. The clinical outcomes of both disease entities are poor with the CML-BP patients being even worse.

Additional possible strategies have been discussed in the literature to distinguish these two entities. One study suggested that CML patients in remission usually have a persistent Ph-positive clone, while Ph-positive ALL patients generally do not have a detectable Ph chromosome during remission [15]. Therefore, evaluation of a remission marrow for the presence of* BCR-ABL1* may be useful. This might be true at the pre-TKIs (tyrosine kinase inhibitor) era. However, at the TKI era, CML patients can reach clinical remission, cytogenetic remission, and molecular remission during which Ph-positive clone or* BCR-ABL* fusion is undetectable, making this strategy impractical. Another study recommended that, by combined morphologic and FISH analysis, the presence of BCR-ABL1 in both the lymphoid and myeloid lineages favors the diagnosis of T-lymphoblastic BP of CML, whereas the presence of BCR-ABL in lymphoid lineage only will favor BCR-ABL-positive T-ALL [8]. However, there are no large series studies regarding the sensitivity and specificity of this strategy. A previous study did show the Ph chromosome in the erythroid and myeloid colonies from 2 Ph-positive ALL patients [16]. The utility of this combined morphologic and FISH analysis strategy needs to be further investigated.

Both of our cases appear to fit into the T-lymphoblastic BP of CML category, but with some unique features. Case 1 is unique in the following: (1) she presented as T-ALL with* BCR-ABL1* rearrangement, but has some concurrent morphologic features of background CML: myeloid hyperplasia with left-shift, mild basophilia, and small, hypolobated megakaryocytes; (2) her relapsed marrow biopsies never showed morphologic features of CML; (3) her remission marrow biopsies never demonstrated features of CML-CP or a karyotype with t(9;22). Case 2 is the first reported T-lymphoblastic BP of CML with a minor BCR breakpoint.

In summary, T-lymphoblastic BP of CML is very rare. Most cases have a history of CML and all cases reported in the literature have a major* BCR* breakpoint in* BCR-ABL1* transcript. Our two cases of T-lymphoblastic BP of CML are very unique. Neither case had a history of CML. One case had never had a CML-CP in the disease course and the other case is the first T-lymphoblastic BP of CML with minor* BCR* breakpoint. Both cases have broadened the spectrum of T-lymphoblastic BP of CML.
